# Delayed cutaneous depigmentation in Vogt–Koyanagi–Harada disease

**DOI:** 10.1093/skinhd/vzag126

**Published:** 2026-08-11

**Authors:** Mohammed Shanshal, Sumbel Khan

**Affiliations:** Department of Dermatology, Imperial College Healthcare NHS Trust, London, UK; Imperial College London, London, UK; Department of Dermatology, Imperial College Healthcare NHS Trust, London, UK; Imperial College London, London, UK

## Abstract

Vogt–Koyanagi–Harada disease may present to dermatologists through delayed integumentary findings after the acute ocular phase. This image-based case shows right frontotemporal depigmentation developing after bilateral panuveitis, with funduscopic changes compatible with convalescent disease.

Dear Editor, A 55-year-old man was evaluated in the dermatology clinic for a stable depigmented patch on the right frontotemporal scalp and forehead that had first appeared in 2022. Examination showed sharply demarcated depigmentation with islands of residual pigmentation (Figure 1a). No other depigmented lesions were present.

**Figure 1:**
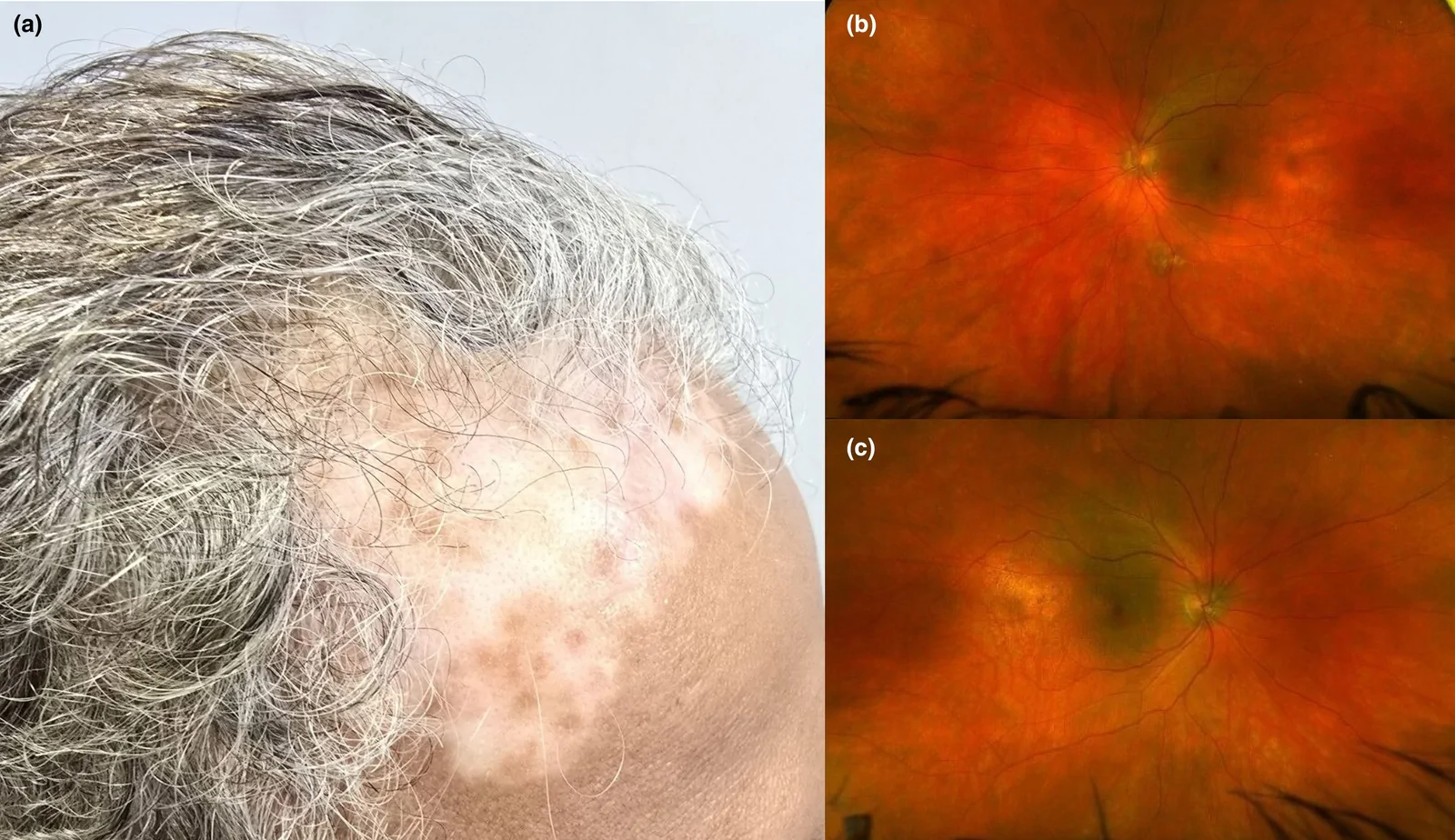
Cutaneous and funduscopic features of Vogt-Koyanagi-Harada disease. (a) Right frontotemporal depigmentation with islands of residual pigmentation. (b, c) Ultra-widefield fundus images showing bilateral chronic chorioretinal depigmentary changes compatible with convalescent Vogt–Koyanagi–Harada disease.

In late 2020 and early 2021, he presented to ophthalmology with painful red eyes, a persistent headache, photophobia and reduced vision; tinnitus and vertigo were denied. Examination showed bilateral panuveitis. Optical coherence tomography showed bilateral subretinal fluid, and retinal imaging showed multifocal chorioretinal inflammation with left optic disc swelling. A clinical diagnosis of Vogt–Koyanagi–Harada (VKH) disease was made. He was treated with systemic glucocorticoids, topical corticosteroid drops and azathioprine, with resolution of subretinal fluid and quiescence of intraocular inflammation. Subsequent ultra-widefield fundus images showed bilateral chronic chorioretinal depigmentary changes compatible with convalescent VKH disease (Figure 1b, c). The delayed cutaneous depigmentation was consistent with integumentary involvement of VKH disease. Dermatological signs in VKH disease can occur in the chronic or convalescent stage and may follow the acute ocular presentation.^1,2^

## Data Availability

Data sharing is not applicable to this article as no datasets were generated or analysed.
